# Hydrocortisone treatment in early sepsis-associated acute respiratory distress syndrome: results of a randomized controlled trial

**DOI:** 10.1186/s13054-016-1511-2

**Published:** 2016-10-15

**Authors:** Surat Tongyoo, Chairat Permpikul, Wasineenart Mongkolpun, Veerapong Vattanavanit, Suthipol Udompanturak, Mehmet Kocak, G. Umberto Meduri

**Affiliations:** 1 Faculty of Medicine, Siriraj Hospital, Mahidol University, No. 2, Prannok Road, Bangkoknoi, Bangkok 10700 Thailand; 2 Faculty of Medicine, Prince of Songkla University, Songkla, Thailand; 3 Department of Preventive Medicine, University of Tennessee Health Science Center, Memphis, TN USA; 4 Division of Pulmonary, Critical Care, and Sleep Medicine, Department of Medicine, Memphis Veterans Affairs Medical Center, Memphis, TN USA

**Keywords:** ARDS, Septic shock, Severe sepsis, Hydrocortisone, Glucocorticoid treatment, Randomized trial

## Abstract

**Background:**

Authors of recent meta-analyses have reported that prolonged glucocorticoid treatment is associated with significant improvements in patients with severe pneumonia or acute respiratory distress syndrome (ARDS) of multifactorial etiology. A prospective randomized trial limited to patients with sepsis-associated ARDS is lacking. The objective of our study was to evaluate the efficacy of hydrocortisone treatment in sepsis-associated ARDS.

**Methods:**

In this double-blind, single-center (Siriraj Hospital, Bangkok), randomized, placebo-controlled trial, we recruited adult patients with severe sepsis within 12 h of their meeting ARDS criteria. Patients were randomly assigned (1:1 ratio) to receive either hydrocortisone 50 mg every 6 h or placebo. The primary endpoint was 28-day all-cause mortality; secondary endpoints included survival without organ support on day 28.

**Results:**

Over the course of 4 years, 197 patients were randomized to either hydrocortisone (*n* = 98) or placebo (*n* = 99) and were included in this intention-to-treat analysis. The treatment group had significant improvement in the ratio of partial pressure of oxygen in arterial blood to fraction of inspired oxygen and lung injury score (*p* = 0.01), and similar timing to removal of vital organ support (HR 0.74, 95 % CI 0.51–1.07; *p* = 0.107). After adjustment for significant covariates, day 28 survival was similar for the whole group (HR 0.80, 95 % CI 0.46–1.41; *p* = 0.44) and for the larger subgroup (*n* = 126) with Acute Physiology and Chronic Health Evaluation II score <25 (HR 0.57, 95 % CI 0.24–1.36; *p* = 0.20). With the exception of hyperglycemia (80.6 % vs. 67.7 %; *p* = 0.04), the rate of adverse events was similar. Hyperglycemia had no impact on outcome.

**Conclusions:**

In sepsis-associated ARDS, hydrocortisone treatment was associated with a significant improvement in pulmonary physiology, but without a significant survival benefit.

**Trial registration:**

ClinicalTrials.gov identifier NCT01284452. Registered on 18 January 2011.

## Background

Acute respiratory distress syndrome (ARDS) is a secondary disease that follows—usually within 6–48 h—a primary disease of multifactorial etiology (most frequently pneumonia and extrapulmonary sepsis [1]) associated with severe systemic inflammation. Inflammatory mediators released into the systemic circulation (systemic inflammation) from the site of infection reach the broad pulmonary capillary surface, producing severe and diffuse inflammatory exudate of the pulmonary lobules and resulting in hypoxemic respiratory failure [2]. In addition to the lung, systemic inflammation involves other vital organs, particularly the cardiovascular and renal systems. In ARDS, baseline plasma inflammatory cytokine levels are associated with a significantly increased risk of death after adjustment for tidal volume (6 ml/kg vs. 12 ml/kg) and severity of illness [3], and higher inflammatory cytokine levels are found in the largest subgroup of sepsis-associated ARDS [3]. Sepsis-associated ARDS, in comparison with sepsis without ARDS [4] or non-sepsis-related ARDS [1, 5], is associated with significantly higher morbidity and higher (up to 60 % [4, 6]) mortality.

In sepsis and ARDS, systemic inflammation is activated by the nuclear factor-κB (NF-κB) signaling system and downregulated by activated glucocorticoid receptor α (GRα) [7]. In these patients, inadequate (endogenous glucocorticoid-activated) GRα-mediated downregulation of proinflammatory transcription factor NF-κB in circulating and tissue cells leads to higher initial levels and persistent elevation over time of plasma and bronchoalveolar lavage markers of inflammation, hemostasis, and tissue repair [7]. Inadequate intracellular GRα-mediated anti-inflammatory activity for the severity of the patient’s illness was recently termed *critical illness-related corticosteroid insufficiency* (CIRCI) [8]. Experimental [9] and clinical research [7] shows that CIRCI can be improved with quantitatively and temporally adequate glucocorticoid administration, and a new meta-analysis provides support for its use in ARDS of multifactorial etiology [10].

Despite the high prevalence and mortality of sepsis-associated ARDS, a prospective randomized controlled trial (RCT) investigating glucocorticoid treatment is lacking. We conducted this RCT to test the hypothesis that the anti-inflammatory action of 7 days of hydrocortisone treatment would result in accelerated disease resolution and lower mortality. The primary outcome was 28-day all-cause mortality; secondary endpoints included 28-day survival without organ support and 60-day mortality.

## Methods

### Study design

We conducted an investigator-initiated, single-center, double-blind, randomized, parallel-groups, placebo-controlled trial between December 2010 and December 2014 in the medical intensive care unit of Siriraj Hospital, Bangkok, Thailand. This trial is registered with ClinicalTrials.gov (NCT01284452). The study was conducted in accordance with the original protocol. The full study protocol is available by contacting the principal investigator.

### Patients

We prospectively screened patients aged 18 years or older meeting the criteria for severe sepsis or septic shock [11]. Patients with severe sepsis or septic shock receiving mechanical ventilation (MV) for hypoxemic respiratory failure were eligible if, within 12 h of study entry, they met the diagnostic criteria for acute lung injury-ARDS according to the American-European Consensus definition [12], as later reclassified on the basis of the 2012 Berlin criteria for the diagnosis of ARDS [13]. Informed consent was obtained from the patients or their legally authorized representatives prior to enrollment. Exclusion criteria included moribund state (i.e., not expected to live more than 24 h), advanced malignancy with life expectancy <6 months, pregnancy, immunosuppressive therapy, underlying disease requiring long-term glucocorticoid treatment within the last 6 months or short-term glucocorticoid treatment within the past 4 weeks, and difficult-to-control diabetes.

### Randomization and study intervention

The coinvestigators (CP, WM, VV) evaluated patients for eligibility, obtained informed consent, and enrolled the participants. After inclusion, patients were randomly assigned without restriction in a 1:1 ratio (hydrocortisone to placebo) according to a computer-generated randomization table derived from www.randomization.com by the principal investigator, who had no role in patient management. A research nurse not otherwise involved in the study prepared both the study drug and placebo. The attending physicians, nursing care teams, research investigators, and participants and their family members were blinded to treatment allocation. Hydrocortisone was given daily as an intravenous bolus (50 mg in 10 ml of normal saline) every 6 h for 7 days. The control group received a comparable volume of normal saline on the same time schedule. Fluid resuscitation and vasopressor treatment were administered according to the Surviving Sepsis Campaign guidelines [14, 15]. Clinicians were asked to comply with the ARDS Network guidelines on low tidal volume and positive end-expiratory pressure [16]. The attending physician managed adjustments of ventilator settings, antibiotic selection, nutritional support, glycemic control, and the initiation of renal replacement therapy.

### Study endpoints and data collection

This RCT tested the hypothesis that the addition of hydrocortisone treatment to standard care would result in accelerated disease resolution and lower mortality. The primary endpoint of the study was all-cause mortality at study day 28. Secondary outcomes included (1) patients alive without organ support (ventilator, renal replacement therapy, and vasopressors) on day 28, (2) MV-free days up to day 28, (3) vasopressor (dopamine, norepinephrine, or adrenaline)-free days up to day 28 (free days to day 28 for patients who died before or on study day 28 was set to 0), and (4) mortality at study day 60. Return to MV after initial successful (off or at least 48 h) extubation was included in the total duration of MV until study day 28.

Baseline data collected included, age, sex, comorbidities, sepsis severity, source of infection, Acute Physiology and Chronic Health Evaluation (APACHE) II score [17], Sequential Organ Failure Assessment score [18], and Lung Injury Score (LIS) [19]. Progression of ARDS was monitored with longitudinal measurements of the LIS (days 0–7 and 14) [19]. For adverse events, we monitored for nosocomial infections, hyperglycemia (glucose >150 mg/dl), and gastrointestinal bleeding until study day 28.

### Statistical analysis

The study protocol stated that the number needed to enroll in this study was calculated by assuming that the 28-day mortality of severe sepsis or septic shock-associated ARDS was 60 % [4, 6]. To detect a 20 % absolute mortality reduction, enrollment of at least 194 patients was required to provide study power of 80 % and a two-sided alpha error of 0.05. There was no planned interim analysis.

The study was analyzed on an intention-to-treat basis. The continuous variables were compared at two or more levels of a categorical factor of interest using the Wilcoxon-Mann-Whitney test, which is a nonparametric counterpart of the two-samples *t* test. The categorical variables were compared with the use of the chi-square test or Fisher’s exact test, when suitable. The primary outcome was evaluated by chi-square test, and the secondary outcomes were analyzed by chi-square test and Wilcoxon-Mann-Whitney test. For the survival analysis, time to an event of interest was calculated from the date of randomization to the date of the event. Patients who did not experience the event were censored at their last-follow up date or at a specified cutoff date such as day 28 or day 60. Survival distributions were estimated using the Kaplan-Meier method and compared by log-rank test.

We also compared the distribution of the time to event, where an event was defined as death or removal of vital organ support independently, by the following prespecified variables: age (≤65 years or >65 years), APACHE II score (<25 or ≥25), LIS (<2.5 or ≥2.5), sepsis severity (absence of shock or vasopressor-dependent shock), ARDS severity (mild or moderate to severe), source of infection (pneumonia or extrapulmonary), and number of comorbidities (two or fewer or three or more). Association of the survival likelihood of a given event (death or removal from organ support, independently) with a set of variables was estimated using a Cox proportional hazards model in which treatment was kept in such multiple regression models regardless of its significance. To describe and compare the profiles of longitudinal variables of interest, a random coefficient modeling framework was used in which time was the primary predictor and time × treatment interaction indicated the treatment differences in the profiles under investigation. A *p* value <0.05 was considered statistically significant for all comparisons. SAS® version 9.4 software (SAS Institute, Cary, NC, USA) was used to conduct all of the analyses.

## Results

**Fig. 1:**
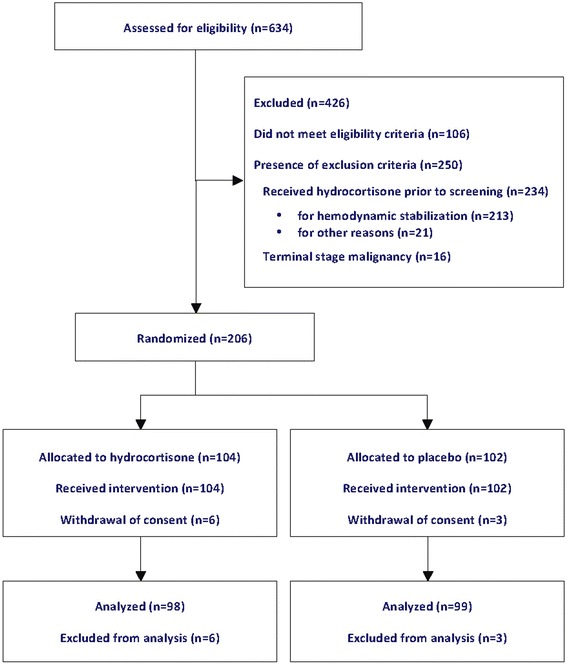
Flow diagram of the progress of the trial

Figure 1 shows progress through the phases of the trial. The trial was stopped when target recruitment was achieved. Of the 206 randomized patients, 9 (6 hydrocortisone, 3 placebo) withdrew consent after randomization and were excluded from analysis. Two hundred thirteen patients were excluded because they were already receiving hydrocortisone for hemodynamic stabilization of refractory septic shock on high-dose vasopressors. The intention-to-treat analysis included 197 patients (98 hydrocortisone, 99 placebo). For the primary endpoint, none of the patients in the intention-to-treat analysis were lost to follow-up. Unless specified otherwise, comparison between groups is reported as hydrocortisone vs. placebo. The mean time (in hours) from meeting severe sepsis criteria to randomization was similar between groups (11.9 ± 7.9 h vs.14.0 ± 10.2 h; *p* = 0.21).

**Fig. 2:**
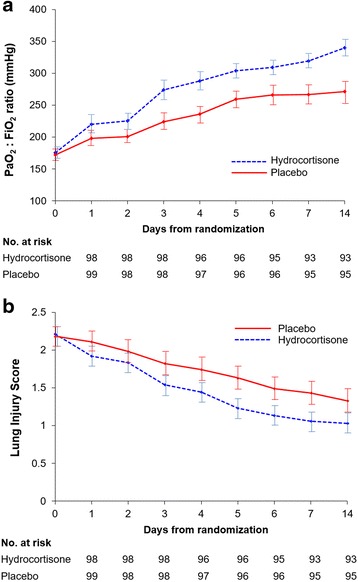
**a** Changes in ratio of partial pressure of oxygen in arterial blood fraction of inspired oxygen (PaO_2_/FiO_2_) over the course of 7 days and on day 14. In comparison with the placebo group, the hydrocortisone group had a significantly higher PaO_2_/FiO_2_ ratio by day 3 (*p* = 0.03) and throughout the observation period. **b** Changes in Lung Injury Score over the course of 7 days and on day 14. In comparison with the placebo group, the hydrocortisone group had a significantly lower Lung Injury Score by day 3 (*p* = 0.003) and throughout the observation period

The baseline characteristics of each group at study entry were similar (Table 1). Most patients had vasopressor-dependent shock (*n* = 154; all but 7 with a lactate level >2 mmol/L) [20] and met criteria for moderate to severe ARDS (*n* = 135). Lactate level (3.9 ± 2.2 mmol/L vs. 4.0 ± 2.5 mmol/L; *p* = 0.95) and distribution of patients with fluid-responsive shock was similar (6.1 % vs. 4.0 %; *p* = 0.55). The most common comorbidities were hypertension and diabetes mellitus. The leading cause of sepsis was pneumonia; overall, bacteremia was present in 28 %. During treatment intervention, the treated group had significant improvement in gas exchange and LIS (Fig. 2), as well as a similar rate of extubation (43.3 % vs. 31.6 %; *p* = 0.11). On study day 7 (mean ± SE), the ratio of partial pressure of oxygen in arterial blood to fraction of inspired oxygen (PaO_2_/FiO_2_) was 319.1 ± 9.7 vs. 266.3 ± 11.7 (*p* = 0.001), and the LIS was 1.1 ± 0.1 vs. 1.4 ± 0.1 (*p* = 0.01). These positive effects persisted at day 14 (last measurement), 7 days after removal of the study drug. In addition, the random coefficients models using the longitudinal data for PaO_2_/FiO_2_ and LIS revealed that the treatment arm had significantly improved profiles over time (*p* = 0.002).

**Table 1:** Baseline characteristics

Characteristics	Hydrocortisone (*n* = 98)	Placebo (*n* = 99)	*p* Value^a^
Age, years	64.5 ± 17.3	64.3 ± 16.0	0.76
Male sex, *n* (%)	50 (51)	51 (51.5)	1.0
APACHE II score^b^	21.7 ± 5.7	21.9 ± 5.7	0.79
SOFA score^c^	10.9 ± 3.5	10.8 ± 3.6	0.91
Lung Injury Score,^d^ mean ± SE	2.2 ± 0.9	2.2 ± 1.0	0.7
Vasopressor-dependent shock, *n* (%)	78 (79.6)	76 (76.8)	0.55
Lactate, mmol/L	3.9 ± 2.2	4.0 ± 2.5	0.95
Lactate >2 mmol/L, *n* (%)	85 (86.7)	82 (82.7)	0.55
Mild ARDS: PaO_2_/FiO_2_ 201–300,^e^ *n* (%)	32 (32.7)	33 (33.7)	1.0
Moderate ARDS: PaO_2_/FiO_2_ 101–200,^e^ *n* (%)	54 (55.1)	48 (48)	0.39
Severe ARDS: PaO_2_/FiO_2_ ≤ 100,^e^ *n* (%)	12 (12.2)	18 (18.4)	0.32
Tidal volume, ml/kg of predicted body weight	7.06 ± 1.12	7.57 ± 1.34	0.04
Tidal volume >8 ml/kg of predicted body weight, *n* (%)	35 (35.4)	41 (41.8)	0.38
Positive end-expiratory pressure, cmH_2_O	7.3 ± 3.0	6.8 ± 2.5	0.59
PaO_2_/FiO_2_, mmHg, mean ± SE	175.4 ± 6.9	172.4 ± 6.7	0.87
Source of infection, *n* (%)			
Pneumonia	49 (50)	51 (52)	0.89
Urinary tract infection	18 (18.4)	19 (19.2)	1.0
Skin and soft tissue infection	15 (15.3)	12 (12.1)	0.54
Intra-abdominal infection	15 (15.3)	7 (7.1)	0.07
Hemoculture-positive	28 (28.6)	28 (28.3)	1.0
Number of comorbidities, median (range)	2 (0–5)	2 (0–5)	1.0
Hypertension	44 (44.9)	43 (43.4)	0.89
Diabetes mellitus	45 (45.9)	37 (37.4)	0.25
Coronary artery disease	18 (18.4)	17 (17.2)	0.85
Stroke	21 (21.4)	14 (14.1)	0.2
Chronic kidney disease	12 (12.2)	12 (12.1)	1.0
Chronic lung disease	11 (11.2)	11 (11.1)	1.0
Cancer and/or immunosuppression	18 (18.4)	26 (26.3)	0.23

*Abbreviations: APACHE II* Acute Physiology and Chronic Health Evaluation II, *ARDS* Acute respiratory distress syndrome, *FiO*_*2*_ Fraction of inspired oxygen, *PaO*_*2*_ Partial pressure of oxygen in arterial blood, *SOFA* Sequential Organ Failure Assessment

Unless specified otherwise, data are reported as mean ± SD

^a^Fisher’s exact test and the Wilcoxon-Mann-Whitney test were used for categorical and continuous variables, respectively

^b^APACHE II score, a severity-determining score, ranges from 0 to 71. The higher scores represent more severe disease

^c^SOFA score ranges from 0 to 24. The higher scores represent more organ failure

^d^The Lung Injury Score ranges from 0 to 4. Scores of 0.1–2.5 indicate mild to moderate lung injury; scores >2.5 indicate severe lung injury

^e^Diagnosis of ARDS according to the Berlin Definition 2012

**Fig. 3:**
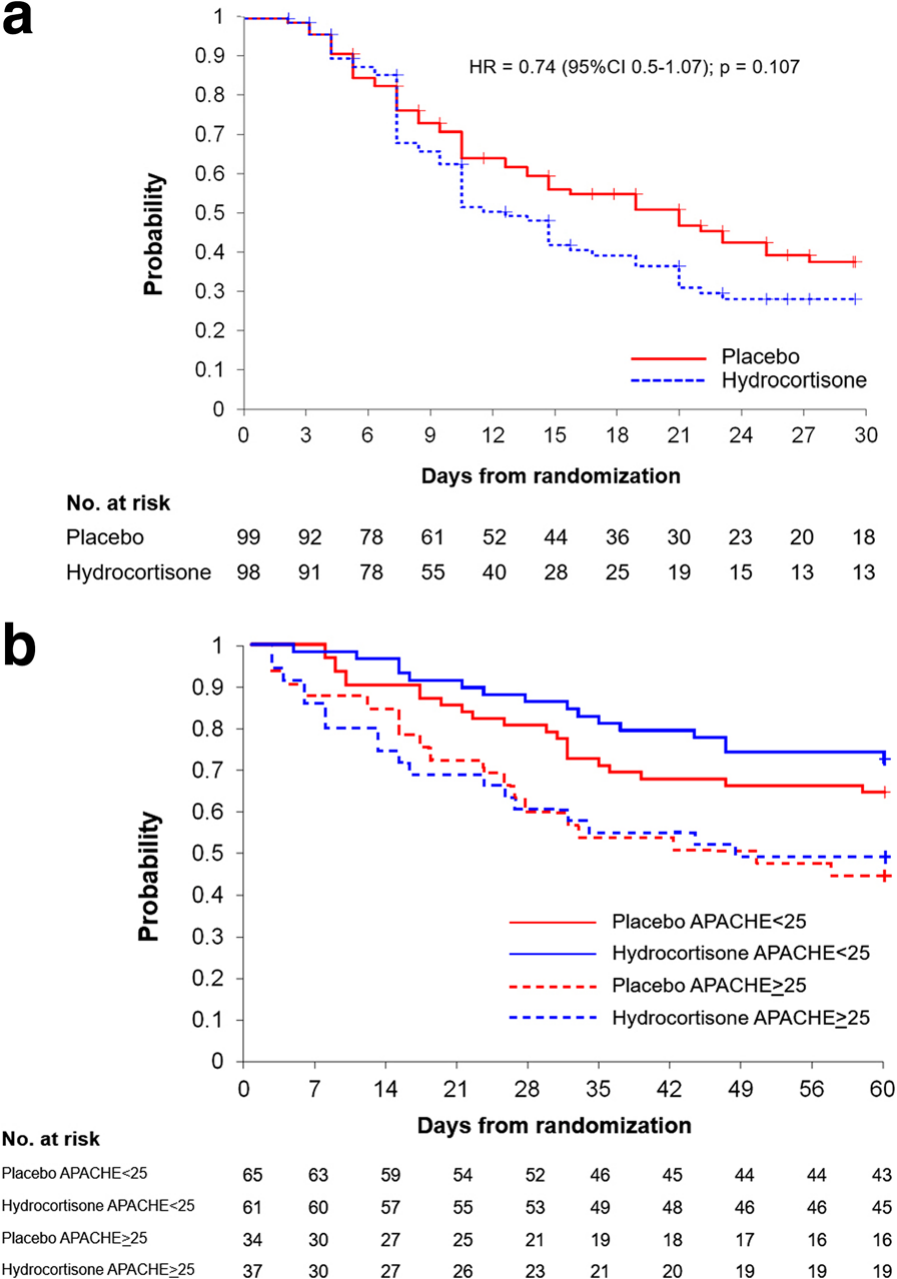
**a** Kaplan-Meier estimate of time to removal of organ support by treatment arm after adjustment by Cox regression analysis for Acute Physiology and Chronic Health Evaluation II (APACHE II) score and Lung Injury Score. *Continuous line* corresponds to the placebo group, and *dashed line* corresponds to the hydrocortisone group. **b** Kaplan-Meier curve illustrates the probability of survival at 60 days based on APACHE II score <25 vs. ≥25. *Continuous line* corresponds to the subgroup with APACHE II score <25 (*blue* = hydrocortisone, *red* = placebo). *Dashed line* corresponds to the subgroup with APACHE score ≥25 (*blue* = hydrocortisone, *red* = placebo). After adjustment for the presence of vasopressor-dependent shock and Lung Injury Score, in the subgroup with APACHE score <25 (*n* = 126), the HR for probability of survival at 28 days was 0.57 (95 % CI 0.24–1.36; *p* = 0.20), and at 60 days it was 0.69 (95 % CI 0.36–1.31; *p* = 0.25). In the subgroup with APACHE II score ≥25 (*n* = 71), the HR for probability of survival at 28 days was 1.05 (95 % CI 0.49–2.23; *p* = 0.60), and at 60 days it was 0.97 (95 % CI 0.50–1.88; *p* = 0.92)

Data for the primary and secondary outcomes are shown in Table 2. By day 28, the treated group had a nonsignificant reduction in duration (days) of MV (10.4 ± 9.4 days vs. 12.4 ± 11.0 days; *p* = 0.16) and vasopressor support (4.8 ± 3.0 days vs. 6.8 ± 5.7 days; *p* = 0.16), as well as a nonsignificant increase in patients alive on day 28 without organ support. To evaluate time to survival without organ support at 28 days, a Kaplan-Meier curve was plotted (Fig. 3a). After adjustment by Cox regression analysis for two covariates found to be significant in the multivariate survival model (Table 3), the hydrocortisone cohort showed an HR of 0.74 (95 % CI 0.51–1.07; *p* = 0.107).

**Table 2:** Primary and secondary outcomes

	Hydrocortisone (*n* = 98)	Placebo (*n* = 99)	Relative risk (95 % CI)	*p* Value^a^
Primary outcome				
Mortality at 28 days, *n* (%)	22 (22.5)	27 (27.3)	0.82 (0.50–1.34)	0.51
Secondary outcomes				
Mortality at 60 days, *n* (%)	34 (34.7)	40 (40.4)	0.86 (0.60–1.23)	0.46
Duration of mechanical ventilation up to day 28, days	11.8 ± 7.8	13.9 ± 9.0		0.17
Mechanical ventilation-free days to day 28	12.0 ± 9.7	9.7 ± 10.0		0.17
Duration of vasopressor treatment,^b^ days	4.8 ± 3.0	6.8 ± 5.7		0.16
Renal replacement therapy, *n* (%)	22 (22.4)	22 (22.2)	1.01 (0.86–1.16)	1.00
Duration of renal replacement therapy dependent,^c^ days	8.1 ± 6.6	8.2 ± 5.2		0.94
Alive on day 28 without organ support, *n* (%)	64 (65.3)	55 (55.6)	1.18 (0.94–1.48)	0.19
Organ support-free days to day 28^d^	11.9 ± 9.7	9.5 ± 9.8		0.13

Unless specified otherwise, data are reported as mean ± SD

^a^For continuous variables, *p* values were calculated using the Wilcoxon-Mann-Whitney test

^b^Data calculated for the patients who received vasopressors

^c^Data calculated for the patients who received renal replacement therapy

^d^Organ support includes the use of mechanical ventilation, vasopressors, or renal replacement therapy

**Table 3:** Univariable and multivariable survival models for time to organ support removal and overall survival on day 28

Covariate at study entry	Time to vital organ support removal		Day 28 survival	
	HR (95 % CI)	*p* Value	HR (95 % CI)	*p* Value
Univariable models				
Hydrocortisone vs. placebo	1.35 (0.93–1.92)	0.11	0.82 (0.47–1.43)	0.49
APACHE II score: <25 vs. ≥25	1.46 (0.97–2.18)	0.066	0.39 (0.22–0.69)	0.0011
Lung Injury Score: <2.5 vs. ≥2.5	1.57 (1.08–2.30)	0.019	0.71 (0.40–1.24)	0.23
Vasopressor-dependent shock: absent vs. present	1.63 (1.09–2.45)	0.018	0.46 (0.19–1.07)	0.071
ARDS severity: mild vs. moderate to severe	0.79 (0.54–1.16)	0.23	1.54 (0.81–2.96)	0.19
Number of comorbidities: ≤2 vs. >2	0.95 (0.64–1.40)	0.79	1.25 (0.71–2.19)	0.44
Source of infection: pneumonia vs. extrapulmonary source	1.09 (0.64–1.85)	0.75	0.90 (0.51–1.58)	0.71
Age: ≤65 years vs. >65 years	1.40 (0.97–2.02)	0.071	1.33 (0.76–2.35)	0.32
Diabetes mellitus: no vs. yes	0.95 (0.64–1.40)	0.79	1.66 (0.84–3.28)	0.18
Hyperglycemia: no vs. yes	0.97 (0.66–1.42)	0.8	1.37 (0.67–2.80)	0.45
Multivariable models				
Hydrocortisone vs. placebo	1.35 (0.93–1.96)	0.107	0.80 (0.46–1.41)	0.44
APACHE II score: <25 vs. ≥25	Not included in multivariable model		0.42 (0.24–0.75)	0.003
Vasopressor-dependent shock: absent vs. present	1.562 (1.04–2.35)	0.032	0.55 (0.23–1.29)	0.17^a^
Lung Injury Score: <2.5 vs. ≥2.5	1.633 (1.11–2.4)	0.012	Not included in multivariable model	

*APACHE II* Acute Physiology and Chronic Health Evaluation II, *ARDS* Acute respiratory distress syndrome

Multivariable survival models for time to survival on day 60 were significant for APACHE II score (HR 0.56, 95 % CI 0.35–0.89; *p* = 0.015) and Lung Injury Score (HR 0.62, 95 % CI 0.39–0.99; *p* = 0.045), and they showed a nonsignificant favorable trend for hydrocortisone vs. placebo (HR 0.81, 95 % CI 0.51–1.28; *p* = 0.37)

^a^This variable was included because it was significant in the day 60 survival model

Unadjusted mortality at day 28 and day 60 was similar (Table 2). Subgroup analysis for the relative risk of death at days 28 and 60 is shown in Table 4. After adjustment by Cox regression analysis for covariates we found significant in the multivariate survival model (Table 3), the HRs for death at days 28 and 60 were 0.80 (95 % CI 0.46–1.41; *p* = 0.44) and 0.81 (95 % 0.51–1.28; *p* = 0.37), respectively. The APACHE II score was the most significant (*p* = 0.001) variable detected in the survival models (Table 3). A Kaplan-Meier curve adjusted for vasopressor-dependent shock and LIS at study entry was generated for the subgroups of patients with APACHE II score <25 vs. ≥25 by treatment (Fig. 3b). After adjustment for the presence of vasopressor-dependent shock and LIS, in the largest subgroup with APACHE II score <25 (*n* = 126), the HR for probability of survival at 28 days was 0.57 (95 % CI 0.24–1.36, *p* = 0.20), and at 60 days it was 0.69 (95 % CI 0.36–1.31; *p* = 0.25). In the subgroup with APACHE II score ≥25 (*n* = 71), the HR for probability of survival at 28 days was 1.05 (95 % CI 0.49–2.23; *p* = 0.60), and at 60 days it was 0.97 (95 % CI 0.50–1.88; *p* = 0.92).

**Table 4:** Subgroup analysis of the risk of death in the hydrocortisone and placebo groups at days 28 and 60

	Day 28				Day 60			
Subgroup	Hydrocortisone (*n* = 98)	Placebo (*n* = 99)	Relative risk with hydrocortisone (95 % CI)	*p* Value	Hydrocortisone (*n* = 98)	Placebo (*n* = 99)	Relative risk with hydrocortisone (95 % CI)	*p* Value
Age								
Age ≤65 years, *n*/total (%)	12/50 (24.0)	16/50 (32.0)	0.75 (0.40–1.42)	0.50	18/50 (36.0)	19/50 (38.0)	0.95 (0.57–1.58)	1.00
Age >65 years, *n*/total (%)	10/48 (20.8)	11/49 (22.5)	0.93 (0.43–1.98)	1.00	16/48 (33.3)	21/49 (42.9)	0.78 (0.47–1.3)	0.40
APACHE II score								
APACHE II score <25, *n*/total (%)	8/61 (13.1)	14/65 (21.5)	0.61 (0.27–1.35)	0.25	16/61 (26.2)	22/65 (33.8)	0.61 (0.27–1.35)	0.44
APACHE II score ≥ 25, *n*/total (%)	14/37 (37.8)	13/34 (38.2)	0.99 (0.55–1.79)	1.00	18/37 (48.7)	18/34 (52.9)	0.92 (0.58–1.45)	0.81
Lung injury score								
Lung injury score < 2.5, *n*/total (%)	11/54 (20.4)	13/58 (22.4)	0.91 (0.45–1.85)	0.82	16/54 (29.6)	17/58 (29.3)	1.01 (0.60–1.79)	1.00
Lung injury score ≥ 2.5, *n*/total (%)	11/44 (25)	13/40 (32.5)	0.77 (0.39–0.52)	0.48	18/44 (40.9)	22/40 (55.0)	0.74 (0.47–1.17)	0.27
Sepsis severity								
Severe sepsis without shock, *n*/total (%)	3/20 (15.0)	3/23 (13.0)	1.15 (0.26–5.07)	1.00	4/20 (20.0)	5/23 (21.7)	0.92 (0.29–2.97)	1.00
Vasopressor dependent shock, *n*/total (%)	19/78 (24.4)	24/76 (31.6)	0.77 (0.46–1.29)	0.37	30/78 (38.5)	35/76 (46.1)	0.84 (0.58–1.21)	0.41
ARDS severity								
Mild ARDS, *n*/total (%)	5/30 (16.7)	7/32 (21.9)	0.76 (0.27–2.14)	0.75	9/30 (30.0)	10/32 (31.3)	0.96 (0.45–2.03)	1.00
Moderate to severe ARDS, *n*/total (%)	17/68 (25.0)	20/67 (29.9)	0.84 (0.48–1.45)	0.57	25/68 (36.8)	30/67 (44.8)	0.82 (0.55–1.24)	0.38
Source of infection								
Pneumonia, *n*/total (%)	11/49 (22.5)	13/51 (25.5)	0.88 (0.44–1.77)	0.82	18/49 (36.7)	19/51 (37.3)	0.99 (0.59–1.64)	1.00
Others, *n*/total (%)	1/10 (10.0)	6/18 (33.3)	0.30 (0.04–2.15)	0.36	1/10 (10.0)	7/18 (38.9)	0.26 (0.04–1.80)	0.19
Number of comorbidities								
≤ 2 comorbidities, *n*/total (%)	18/67 (26.9)	22/77 (28.6)	0.94 (0.55–1.60)	0.85	25/67 (37.3)	30/77 (39.0)	0.96 (0.63–1.45)	0.87
≥ 3 comorbidities, *n*/total (%)	4/31 (12.9)	5/22 (22.7)	0.57 (0.17–1.88)	0.46	9/31 (29.0)	10/2 (45.5)	0.64 (0.31–1.31)	0.26

*APACHE II* Acute Physiology and Chronic Health Evaluation II, *ARDS* Acute respiratory distress syndrome

With the exception of hyperglycemia (80.6 % vs. 67.7 %; *p* = 0.04), the rate of adverse events was similar (Table 5). The incidence of hyperglycemia in patients with (80 % vs. 70 %; *p* = 0.45) and without (81 % vs. 66 %; *p* = 0.09) underlying diabetes was similar. Neither diabetes nor hyperglycemia (at study entry or after randomization) had an impact on survival (data not shown).

**Table 5:** Adverse events

Event	Hydrocortisone (*n* = 98)	Placebo (*n* = 99)	*p* Value
Nosocomial infection	17 (17.3)	19 (19.2)	0.74
Lung infection	10 (10.2)	12 (12.1)	0.67
Catheter-related bloodstream infection	3 (3.1)	5 (5.1)	0.72^a^
Urinary tract infection	3 (3.1)	2 (2)	0.68^a^
Other nosocomial infection	1 (1)	3 (3)	0.62^a^
Hyperglycemia (glucose >150 mg/dl)	79 (80.6)	67 (67.7)	0.038
New-onset atrial fibrillation	3 (3.1)	5 (5.1)	0.72
Reintubation within 28 days	6 (6.1)	7 (7.2)	0.78
Gastrointestinal bleeding	3 (3.1)	4 (4)	1.00

^a^Fisher’s exact test *p* values due to small cell counts

## Discussion

This is the first prospective trial investigating a 7-day course of low-dose hydrocortisone treatment in patients with sepsis-associated early ARDS. Hydrocortisone treatment was associated with rapid and sustained improvement in pulmonary physiology and a nonsignificant increase (65.3 % vs. 55.6 %; *p* = 0.19) in patients alive on day 28 without organ support. The significant physiological improvements did not translate into an overall survival benefit for the whole group (HR 0.80, 95 % CI 0.46–1.41; *p* = 0.44), however, and for the largest subgroup of patients with APACHE II score <25 (HR 0.57, 95 % CI 0.24–1.36; *p* = 0.20). Importantly, in agreement with the literature on sepsis [21] and ARDS [10], we did not find an increased risk of complications, with the exception of hyperglycemia, and hyperglycemia did not affect outcome. Our findings are placed in the context of the limitations of the trial and the updated literature.

Our study has several limitations. First, and most important, our study was significantly underpowered by the recruitment of patients with less severe illness and lower mortality. While the original sample size calculation was based on a control mortality of 60 %, originating from the findings of the largest prior trial [6], our control 28-day mortality was almost half that and similar to the most recent report [5]. This is partly attributable to two main factors: (1) exclusion of a large number of patients who, during the screening process, were already receiving hydrocortisone treatment for refractory septic shock [14]; and (2) improved outcome as a result of implementing the international guidelines of the Surviving Sepsis Campaign [14]. In comparison with prior RCTs, our trial differs by not including longitudinal measurements of systemic inflammatory response syndrome parameters or markers of systemic inflammation [6, 22–27] and an assessment of adrenal function [6, 26].

Seven RCTs published in the English language investigated prolonged glucocorticoid treatment in ARDS [6, 22–27]. In each trial, glucocorticoid treatment was associated with a significant reduction in markers of systemic inflammation (inflammatory cytokines and/or C-reactive protein levels) and improvement in lung function. Three trials investigated hydrocortisone treatment in sepsis [22, 23] or septic shock-associated ARDS [6]. The largest study [6] is a post hoc analysis of 177 patients with ARDS from a trial of vasopressor-dependent septic shock investigating hydrocortisone (dosage similar to our trial) and fludrocortisone (50 μg daily) treatment [28]. In the largest trial [6], among those randomized to receive the active drug, the larger subgroup (*n* = 129) of nonresponders to a short corticotropin test, contrary to responders (*n* = 48), had more days alive and off a mechanical ventilator (5.6 ± 8.8 days vs. 2.6 ± 6.6 days; *p* = 0.006) and improved survival (47 % vs. 25 %; *p* = 0.021). Two other trials [22, 23] of patients with severe community-acquired pneumonia included 94 patients on MV with early ARDS (16 % with shock). In both trials [22, 23], a hydrocortisone bolus (200 mg) followed by an infusion (240–300 mg/24 h) was associated with a significant reduction in duration of MV; one research group reported improved survival [22].

Four trials investigated prolonged methylprednisolone treatment in early [26, 27] and late [24, 25] ARDS and were the subject of a recent individual patient data (IPD) meta-analysis (*n* = 322) [10]. By study day 28, the methylprednisolone group had fewer patients dying while on MV (12 % vs. 29 %; *p* < 0.001) and more patients successfully extubated (80 % vs. 50 %; *p* < 0.001); hospital mortality was decreased (20 % vs. 33 %; *p* = 0.006). Importantly, each trial [24–27] consistently reported a 9- to 10-day reduction in duration of MV during methylprednisolone administration (Table S3 supplementary material in [10]). All but one trial [25] incorporated slow tapering of the study drug after extubation; lack of slow tapering contributed to increased return to MV [25].

Our trial is a single-center RCT, which may raise concerns regarding the generalization of the results based on participants’ characteristics and the intervention tested. In comparison with our study, severity of illness scores and LISs were higher in five RCTs [6, 24–27] and similar in two RCTs [22, 23]. Fundamental variables of the treatment protocol that may significantly affect overall response to glucocorticoid treatment in ARDS include study drug, dosage, and duration of administration, including tapering [7]. Our treatment intervention was comparable to the one investigated in the two RCTs with lower severity of illness [22, 23] and differed from the other five RCTs by study drug (hydrocortisone vs. hydrocortisone plus fludrocortisone [6] or methylprednisolone [24–27]), duration of treatment (7 days vs. 14–28 days [24–27]), and tapering (lack of tapering vs. slow tapering [24, 26, 27]).

In the past exogenous glucocorticoids were thought to be qualitatively indistinguishable because they act via the same receptor, but qualitative differences have been discovered, and one glucocorticoid cannot be simply replaced by another [29]. In reference to the RCTs described above [6, 24–27], hydrocortisone has (1) less anti-inflammatory potency when it is not combined with fludrocortisone (a drug with both mineralocorticoid and glucocorticoid activities) and (2) lesser direct inhibitory NF-κB activity than methylprednisolone (half-maximal inhibitory concentration for NF-κB inhibition 16 and 2.8, respectively) [30]. Methylprednisolone, because of its greater penetration in lung tissue and longer residence time [31], is the most frequently used intravenous glucocorticoid for the treatment of severe acute inflammatory lung diseases [32].

New understanding of the central role played by glucocorticoids in resolution of inflammation and restoration of tissue homeostasis [33] provides support for longer duration of treatment incorporating slow tapering (up to 4 weeks). A recent IPD meta-analysis demonstrates that lack of glucocorticoid tapering after extubation of patients with ARDS is frequently associated with clinical deterioration and return to MV [10]. Similarly, researchers in a comparison study of two concurrent RCTs—hydrocortisone 7-day infusion vs. prolonged methylprednisolone—reported that removing hydrocortisone without tapering led to a rebound of systemic inflammation with worsening organ dysfunction requiring reintubation [34]. In our study, we did not measure markers of inflammation and cannot comment on the presence of rebound inflammation. After completion of study treatment, however, the hydrocortisone group continued to demonstrate improved gas exchange and LIS, and the two groups had a similar rate of reintubation by day 28. While these findings may suggest that a clinically significant rebound effect is less likely, most patients (*n* = 55) had hydrocortisone treatment discontinued prior to extubation, masking a possible rebound.

Our trial does not confirm the benefits reported on the basis of prior RCTs for reduction in duration of MV and intensive care unit stay [22–27] or survival [22–24, 26, 27]. Our study is underpowered for the exploratory analysis to demonstrate a survival benefit in patients with APACHE II scores <25. It is likely that this cohort had a lesser degree of systemic inflammation than patients with APACHE II scores ≥25. Lacking measurement of markers of systemic inflammation over time, we cannot comment on whether our treatment protocol was less effective in achieving a significant anti-inflammatory effect in those with APACHE II scores ≥25.

## Conclusions

In sepsis-associated ARDS, early administration of hydrocortisone 50 mg intravenously every 6 h for 7 days was safe and associated with improved lung function without a significant survival benefit. Our study is underpowered for the exploratory analysis to demonstrate a survival benefit in patients with APACHE II scores <25.

## Key messages

- Pneumonia and extrapulmonary sepsis are the leading causes of ARDS. Sepsis-associated ARDS, in comparison with sepsis without ARDS or non-sepsis-related ARDS, is associated with higher morbidity and mortality.
- Despite the high prevalence and mortality of sepsis-associated ARDS, a prospective RCT investigating glucocorticoid treatment is lacking. This is the first randomized, double-blind, placebo-controlled trial evaluating the efficacy of hydrocortisone treatment in early sepsis-associated ARDS.
- Hydrocortisone administration for 7 days was associated with improvement in oxygenation parameters and LIS without achieving a significant survival benefit. After hydrocortisone removal, improvements in oxygenation and LIS were maintained. With the exception of hyperglycemia, we found no increased rate of adverse events.

## Abbreviations

APACHE II: Acute Physiology and Chronic Health Evaluation II
ARDS: Acute respiratory distress syndrome
CIRCI: Critical illness-related corticosteroid insufficiency
FiO_2_: Fraction of inspired oxygen
GRα: Glucocorticoid receptor α
IPD: Individual patient data
LIS: Lung Injury Score
MV: Mechanical ventilation
NF-κB: Nuclear factor-κB
PaO_2_: Partial pressure of oxygen in arterial blood
RCT: Randomized controlled trial
SOFA: Sequential Organ Failure Assessment

## References

[1] Rubenfeld GD, Caldwell E, Peabody E, Weaver J, Martin DP, Neff M (2005). Incidence and outcomes of acute lung injury. N Engl J Med.

[2] Schwingshackl A, Meduri GU (2016). Rationale for prolonged glucocorticoid use in pediatric ARDS: what the adults can teach us. Front Pediatr..

[3] Parsons PE, Eisner MD, Thompson BT, Matthay MA, Ancukiewicz M, Bernard GR (2005). Lower tidal volume ventilation and plasma cytokine markers of inflammation in patients with acute lung injury. Crit Care Med.

[4] Mikkelsen ME, Shah CV, Meyer NJ, Gaieski DF, Lyon S, Miltiades AN (2013). The epidemiology of acute respiratory distress syndrome in patients presenting to the emergency department with severe sepsis. Shock.

[5] Sheu CC, Gong MN, Zhai R, Chen F, Bajwa EK, Clardy PF (2010). Clinical characteristics and outcomes of sepsis-related vs non-sepsis-related ARDS. Chest.

[6] Annane D, Sébille V, Bellissant E, Ger-Inf-05 Study Group (2006). Effect of low doses of corticosteroids in septic shock patients with or without early acute respiratory distress syndrome. Crit Care Med.

[7] Meduri GU, Annane D, Chrousos GP, Marik PE, Sinclair SE (2009). Activation and regulation of systemic inflammation in ARDS: rationale for prolonged glucocorticoid therapy. Chest..

[8] Marik PE, Pastores S, Annane D, Meduri G, Sprung C, Arlt W (2008). Clinical practice guidelines for the diagnosis and management of corticosteroid insufficiency in critical illness: recommendations of an international task force. Crit Care Med..

[9] Wang XQ, Zhou X, Zhou Y, Rong L, Gao L, Xu W (2008). Low-dose dexamethasone alleviates lipopolysaccharide-induced acute lung injury in rats and upregulates pulmonary glucocorticoid receptors. Respirology.

[10] Meduri GU, Bridges L, Shih MC, Marik PE, Siemieniuk RA, Kocak M (2016). Prolonged glucocorticoid treatment is associated with improved ARDS outcomes: analysis of individual patients’ data from four randomized trials and trial-level meta-analysis of the updated literature. Intensive Care Med.

[11] Bone RC, Balk RA, Cerra FB, Dellinger RP, Fein AM, Knaus WA (1992). Definitions for sepsis and organ failure and guidelines for the use of innovative therapies in sepsis. Chest.

[12] Bernard GR, Artigas A, Brigham KL, Carlet J, Falke K, Hudson L (1994). The American-European Consensus Conference on ARDS. Definitions, mechanisms, relevant outcomes, and clinical trial coordination. Am J Respir Crit Care Med.

[13] Force ADT, Ranieri VM, Rubenfeld GD, Thompson BT, Ferguson ND, Caldwell E (2012). Acute respiratory distress syndrome: the Berlin Definition. JAMA.

[14] Dellinger RP, Levy MM, Carlet JM, Bion J, Parker MM, Jaeschke R (2008). Surviving Sepsis Campaign: international guidelines for management of severe sepsis and septic shock: 2008. Intensive Care Med.

[15] Dellinger RP, Levy MM, Rhodes A, Annane D, Gerlach H, Opal SM (2013). Surviving Sepsis Campaign: international guidelines for management of severe sepsis and septic shock, 2012. Intensive Care Med.

[16] The Acute Respiratory Distress Syndrome Network (2000). Ventilation with lower tidal volumes as compared with traditional tidal volumes for acute lung injury and the acute respiratory distress syndrome. N Engl J Med..

[17] Knaus WA, Draper EA, Wagner DP, Zimmerman JE (1985). APACHE II: a severity of disease classification system. Crit Care Med.

[18] Vincent JL, de Mendonça A, Cantraine F, Moreno R, Takala J, Suter PM (1998). Use of the SOFA score to assess the incidence of organ dysfunction/failure in intensive care units: results of a multicenter, prospective study. Crit Care Med.

[19] Murray JF, Matthay MA, Luce JM, Flick MR (1988). An expanded definition of the adult respiratory distress syndrome. Am Rev Respir Dis.

[20] Singer M, Deutschman CS, Seymour CW, Shankar-Hari M, Annane D, Bauer M (2016). The Third International Consensus Definitions for Sepsis and Septic Shock (Sepsis-3). JAMA.

[21] Annane D, Bellissant E, Bollaert PE, Briegel J, Keh D, Kupfer Y (2015). Corticosteroids for treating sepsis. Cochrane Database Syst Rev.

[22] Confalonieri M, Urbino R, Potena A, Piattella M, Parigi P, Puccio G (2005). Hydrocortisone infusion for severe community-acquired pneumonia: a preliminary randomized study. Am J Respir Crit Care Med.

[23] Sabry NA, Omar EED (2011). Corticosteroids and ICU course of community acquired pneumonia in Egyptian settings. Pharmacol Pharm.

[24] Meduri GU, Headley AS, Golden E, Carson SJ, Umberger RA, Kelso T (1998). Effect of prolonged methylprednisolone therapy in unresolving acute respiratory distress syndrome: a randomized controlled trial. JAMA.

[25] Steinberg KP, Hudson LD, Goodman RB, Hough CL, Lanken PN, Hyzy R (2006). Efficacy and safety of corticosteroids for persistent acute respiratory distress syndrome. N Engl J Med.

[26] Meduri GU, Golden E, Freire AX, Taylor E, Zaman M, Carson SJ (2007). Methylprednisolone infusion in early severe ARDS: results of a randomized controlled trial. Chest.

[27] Rezk N, Ibrahim A (2013). Effects of methylprednisolone in early ARDS. Egypt J Chest Dis Tuberc.

[28] Annane D, Sébille V, Charpentier C, Bollaert PE, François B, Korach JM (2002). Effect of treatment with low doses of hydrocortisone and fludrocortisone on mortality in patients with septic shock. JAMA.

[29] Czock D, Keller F, Rasche FM, Häussler U (2005). Pharmacokinetics and pharmacodynamics of systemically administered glucocorticoids. Clin Pharmacokinet.

[30] Li S, Kang GS, Miller DD, Meduri GU, Yates CR (2003). Evaluation of AP-1 and NF-κB inhibitory potency for oral glucocorticoids. AAPS PharmSci.

[31] Greos LS, Vichyanond P, Bloedow DC, Irvin CG, Larsen GL, Szefler SJ (1991). Methylprednisolone achieves greater concentrations in the lung than prednisolone: a pharmacokinetic analysis. Am Rev Respir Dis.

[32] Jantz MA, Sahn SA (1999). Corticosteroids in acute respiratory failure. Am J Respir Crit Care Med.

[33] Busillo JM, Cidlowski JA (2013). The five Rs of glucocorticoid action during inflammation: ready, reinforce, repress, resolve, and restore. Trends Endocrinol Metab.

[34] Nawab Q, Golden E, Confalonieri M, Umberger R, Meduri G (2011). Corticosteroid treatment in severe community-acquired pneumonia: duration of treatment affects control of systemic inflammation and clinical improvement. Intensive Care Med..

